# Coupled Effect of Chloride Corrosion and Repeated Uniaxial Compressive Loading on Unsaturated Concrete

**DOI:** 10.3390/ma16082947

**Published:** 2023-04-07

**Authors:** Bing Han, Ziwei Song, Jinquan Zhang, Huibing Xie, Wutong Yan, Yun Liu, Jiaping Yu

**Affiliations:** 1 School of Civil Engineering, Beijing Jiaotong University, Beijing 100044, China; zw.song@bjtu.edu.cn (Z.S.); jq.zhang@rioh.cn (J.Z.); xiehb@bjtu.edu.cn (H.X.); 16121070@bjtu.edu.cn (Y.L.); 19119907@bjtu.edu.cn (J.Y.); 2 Research Institute of Highway Ministry of Transport, Beijing 100088, China; 3 Consulting Department of Bridge and Tunnel, China Railway Economic and Planning Research Institute Co., Ltd., Beijing 100038, China; 15115261@bjtu.edu.cn

**Keywords:** unsaturated concrete, repeated loading, chloride-induced corrosion, moisture diffusion coefficient, chloride diffusion coefficient, theoretical model

## Abstract

Concrete structure performance continuously deteriorates during operation, and the performance is simultaneously affected by chloride corrosion and repeated traffic loading. Repeated-loading-induced cracks have an impact on the rate of chloride corrosion. Chloride-induced concrete corrosion also affect the stress level of the structure under loading. Therefore, the coupled effect of repeated loading and chloride corrosion on the structural performance needs to be investigated. An upgraded test device was developed for chloride corrosion testing of unsaturated concrete structures under repeated loading. Based on the experimental results, considering the influence of repeated loading on the moisture diffusion coefficient and the chloride diffusion coefficient, a chloride transport model for unsaturated concrete under the coupled effect of repeated uniaxial compressive loading and corrosion was established. The chloride concentration under coupled loading was determined by the Crank–Nicolson finite difference method and the Thomas algorithm, and then chloride transport under the coupled effect of repeated loading and corrosion was analyzed. The results showed that the stress level and the repeated loading cycles directly affect the relative volumetric water content and chloride concentration in unsaturated concrete. The effect of chloride corrosion is more severe in unsaturated concrete compared to saturated concrete.

## 1. Introduction

Concrete exposed to the marine environment is susceptible to various physical and chemical degradation processes. After harmful ions penetrate the concrete, the normal service life of the structure will be shortened [1]. Fulin et al. [2] systematically reviewed the influence of the marine environment on the deterioration mechanisms, performance and durability of concrete materials and structures, and analyzed the chloride resistance of cementitious composites [3]. Chloride ions can penetrate concrete exposed to chloride salts by diffusion, desiccation and absorption processes [4,5] and change the internal fine and micro-composition of concrete and affect its performance. Therefore, chloride diffusion is the main factor leading to concrete structural performance deterioration in coastal areas [6,7,8,9].

Some studies have focused on the chloride transport behavior of saturated concrete under repeated loading [10,11,12,13,14,15]. The chloride transport mechanism in saturated concrete is mainly diffusion. Compared with unsaturated concrete, the rate of chloride transfer in saturated concrete is relatively slow. However, chloride ions in the superstructure of the bridge mainly come from salt spray. The environmental factors in the coastal salt-spray zones are complex and have a great influence on the internal microenvironment of concrete. In practical engineering, except for concrete structures in tidal zones and splash zones, concrete is mostly unsaturated during its long service life [16,17]. 

In addition, during operation, concrete structures are subject to continuously repeated loading such as vehicles, wind, waves, etc., which could cause fatigue damage in concrete, increase the connectivity between pores, provide more channels for chloride intrusion, and lead to the corrosion of reinforcements in the structures [10,11,12,13]. The bearing capacity of the structures decreases sharply, which brings serious safety and reliability concerns [14,15]. Thus, it is imperative to study the chloride transport in concrete after fatigue loading.

Chloride ions in unsaturated concrete are continuously transported into concrete by capillary-absorption-induced convection and by concentration-gradient-induced diffusion [18]. The coupling of these two effects intensifies the corrosion of the reinforcements. Zhang [19] proposed an analytical model to predict the relative diffusion coefficient of chloride in cement materials at different moisture concentration levels. Yang [20] divided concrete into two parts, the matrix and microcracks, and established a new theoretical model of chloride transport under fatigue loading. Bao [21] and Wang et al. [22] explored the influence of continuous pressure and tension on chloride transport behavior in unsaturated concrete. Sun [23] established a prediction model of chloride migration in unsaturated concrete, which took both diffusion and convection into account. The diffusion and penetration under drying–wetting cycle conditions were analyzed, and the model was verified through drying–wetting cycle tests. Considering the interaction between water transport and curvature in the mesostructure of concrete, Cao et al. [24] established a variable coefficient constitutive model of chloride transport under the coupling of bending loads and drying–wetting cycles. Chen [25] established a diffusion–convection constitutive equation for chloride in unsaturated concrete by considering the interaction between moisture and chloride migration in concrete and other influences on the chloride diffusion coefficient. However, the majority of research has focused on investigating the influence of the drying–wetting cycle on concrete, while chloride corrosion of concrete under repeated loads in salt spray zones is rarely studied. The transport behavior of moisture and chloride in spray zones is different to that under the drying–wetting cycle. In addition, the influence of the moisture diffusion coefficient and the chloride diffusion coefficient on chloride transport has rarely been considered in the established models, and their relationship has not been studied.

In this paper, chloride transport in unsaturated concrete under the coupled effect of repeated loading and chloride diffusion was investigated. The effects of stress levels and the number of loading cycles on chloride transport were also analyzed. Based on experimental results, a modified chloride transport model of unsaturated concrete was proposed and verified. The proposed model provides a reference for the design, construction, operation, and maintenance of unsaturated concrete structures.

## 2. Experiment Program

### 2.1. Materials and Specimen Preparation

According to the recommended dimensions and shapes of concrete specimens subjected to a coupled compressive loading and chloride penetration. in RILEM TC 246-TDC [26], a total of 11 groups of concrete specimens with sizes 150 mm × 150 mm × 550 mm were chosen. The mix proportions of the concrete are listed in Table 1.

In addition, three cubic concrete specimens (150 mm × 150 mm × 150 mm) and three prismatic concrete specimens (150 mm × 150 mm × 300 mm) were constructed to test their ultimate compressive strength. The average value of the ultimate compressive strength of the prism was used as the basis for calculating the maximum load and the minimum load under uniaxial compression repeated loading. In this test, the ultimate compressive strengths of cubic and prismatic concrete were 47.8 MPa and 32.5 MPa, respectively.

**Figure 1:**
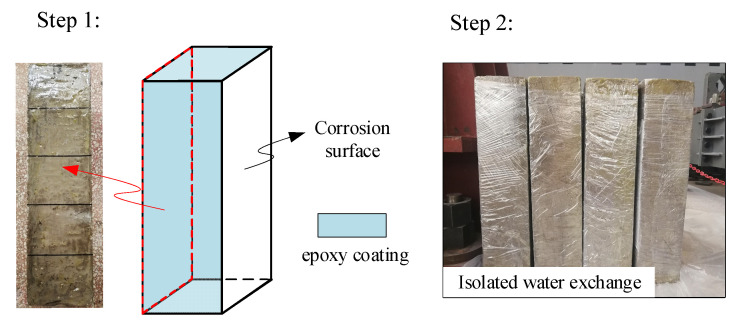
The specimen preparation process.

After 28 days of standard curing, all specimens were placed in a drying system. The initial temperature of the drying system was set to 20 °C. Then, the temperature was increased by 20 °C every two hours until it reached 105 °C [27]. The purpose of this method was to prevent the temperature stress caused by excessive internal and external temperature differences from causing concrete cracking. When the change in specimen weight within 24 h was less than 0.1%, the specimens were considered to have reached a completely dry state [27]. To ensure an identical initial unsaturated state, the concrete specimens were stored in a dryer at 20 °C for an additional 24 h after drying. In the experimental process, to prevent water evaporation or absorption, five surfaces of the concrete specimens were coated with epoxy resin, leaving the test surface uncoated. After the epoxy resin was completely dried, the concrete specimens were wrapped in plastic film and completely isolated from the external environment, as shown in Figure 1.

### 2.2. Testing Device

**Figure 2:**
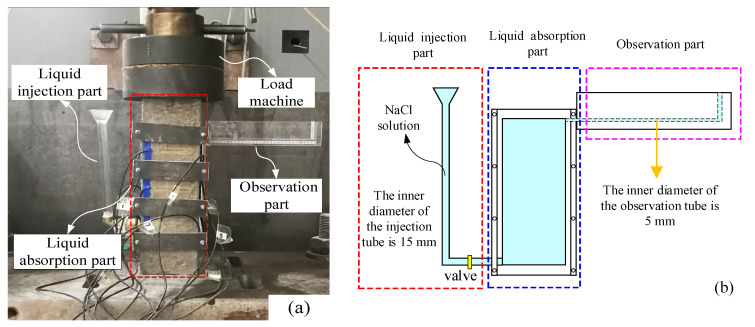
Schematic diagram of testing device: (**a**) test setup and (**b**) schematic diagram.

To measure the liquid absorption rate of unsaturated concrete specimens under repeated uniaxial compressive loading, we referred to the test process of Yan [26], and an improved test device was designed and constructed. The coupled effect of repeated loading and chloride corrosion could be measured, as shown in Figure 2. The concentration of sodium chloride solution was 15%.

The height of the specimens was relatively short; thus, the hydrostatic pressure distribution along the height direction was not significant and could be ignored. In addition, to overcome the influence of liquid gravity on concrete capillary suction, this paper adopted the side suction method. When the valve is closed, the solution in the observed part of the concrete will reduce due to capillary absorption. The amount of liquid absorption of the concrete can be obtained by observing the change in the observed area.

### 2.3. Loading Scheme

To investigate the influence of repeated uniaxial compression loading on chloride transport in unsaturated concrete, factors such as the stress level and repeated loading cycles were studied. The stress levels were set to 0, 0.15, 0.3, and 0.45. The loading cycles were repeated for 50 × 10^4^ cycles, 100 × 10^4^ cycles, 150 × 10^4^ cycles, and 200 × 10^4^ cycles. The loading frequency was 4Hz. The loading method was divided into two types: coupled and uncoupled. Coupled loading means that the test specimens were subjected to both chloride corrosion as well as repeated uniaxial compressive loading. For uncoupled loading, specimens were subjected to a chloride corrosion test after the repeated loading and unloading. The testing scenario is detailed in Table 2. When the uniaxial compression was repeatedly loaded for the specified number of cycles, the loading was stopped.

### 2.4. Test Measurements

#### 2.4.1. Liquid Absorption of Concrete

Sodium chloride solution was added from the liquid injection part of the liquid storage tank until the solution had reached the specified position in the observation tube. Then, the location of the solution in the observation tube was recorded at specified time intervals according to ASTM C1585-04 [28], as shown in Table 3.

#### 2.4.2. Chloride Concentration

**Figure 3:**
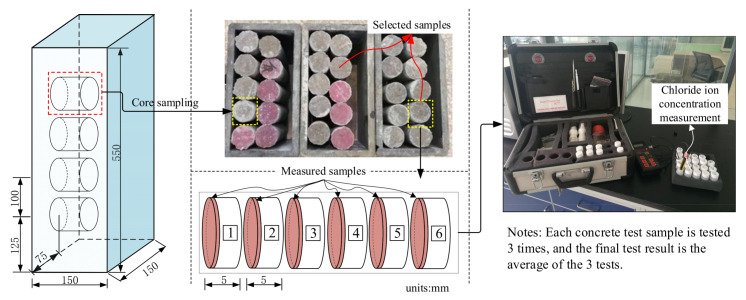
The chloride concentration measurement process.

The concentration of chloride in the unsaturated concrete was determined by the drilling core sampling method and selective electrode method. A GERMANN RCT rapid chlorine meter for concrete was used to measure the chloride concentration. A drill with an inner diameter of 50 mm was used to drill six samples in each test specimen. The drilling depths were 1 mm, 6 mm, 11 mm, 16 mm, 21 mm, and 26 mm. The chloride concentration measurement process is shown in Figure 3.

## 3. Test Results and Discussion

### 3.1. Liquid Absorption of Unsaturated Concrete

**Figure 4:**
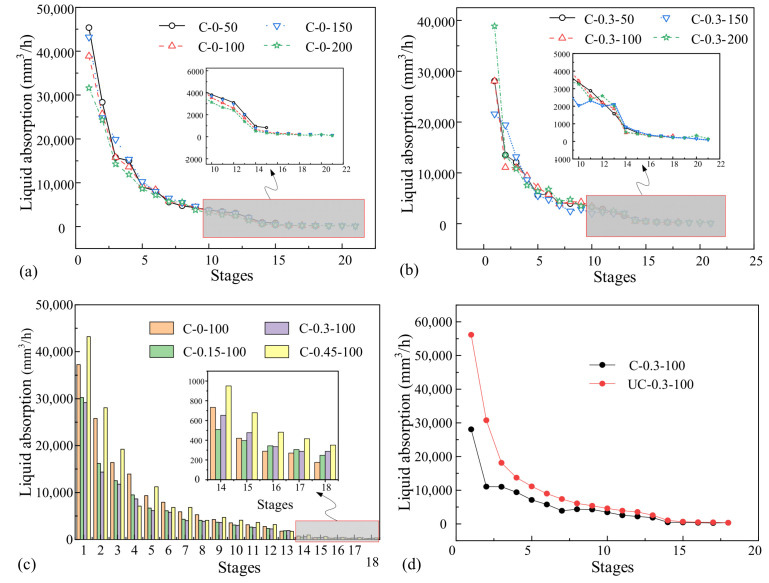
Liquid absorption of unsaturated concrete under different loading scenarios: (**a**) corrosion only; (**b**) different loading cycles; (**c**) different stress levels; and (**d**) coupling and uncoupling.

To reflect the level of liquid absorption of the unsaturated concrete at varying numbers of loading cycles and stress levels, the measured liquid absorption at different stages was converted into absorption per hour. The absorption rate of unsaturated concrete at each loading stage is shown in Figure 4. These curves indicate that liquid absorption of unsaturated concrete declines gradually with absorption stages and finally approaches zero. Regardless of the stress level and loading cycles, the liquid absorption of unsaturated concrete in previous stages of the test is greater than that in later stages. In the early stages of the test, the capillary absorption in unsaturated concrete pores is strong. However, as the absorption time increases, the moisture inside the concrete gradually increases. The suction force of the capillary decreases gradually, resulting in less liquid absorption of the unsaturated concrete at later stages.

Figure 4a,b depicts the variation in the unsaturated concrete liquid absorption rate with corrosion time. Even under different stress levels, the variation in liquid absorption was the same. However, due to the randomness in the initial concrete defects, the initial liquid absorption rate was different. Under different stress levels and loading times of unsaturated concrete, with the increase in suction time, the absorption of liquid decreases rapidly first, then tends to be stable, and finally approaches 0. This indicates that significant convection occurs in the unsaturated concrete at the initial stage of suction. After approaching saturation, the convection weakens and the transmission mode of the liquid is diffusion.

As shown in Figure 4c, after stage 5, the difference in the liquid absorption rate decreases and an increase in corrosion time has little effect on the liquid absorption of the concrete. In stages 1–5, the liquid absorption of test specimen C-0-100 in each stage is significantly larger than C-0.15-100 and C-0.3-100. Liquid absorption of the test specimen C-0.3-100 is the lowest. When the stress level is less than 0.3, the liquid absorption of unsaturated concrete decreases with the increase in stress levels. This is due to the compaction effect created by the low stress. At later stages, the liquid absorption of test specimens C-0.15-100 and C-0.3–100 is greater than C-0-100 at each stage. This is closely related to the initiation and propagation of micro-cracks in concrete. External fatigue loadings with high stress levels are more likely to accelerate micro-crack initiation, which accelerates the liquid transport in the concrete.

As shown in Figure 4d, except for the last few stages, the damage inside the uncoupled loading specimens is larger than that inside the coupled loading specimens. This is mainly because the internal damage of the coupled loading specimens increases with the increase in loading cycles. However, the internal damage of the uncoupled loading specimens already reached the final damage state of the coupled loading specimens before the chloride diffusion test.

### 3.2. Liquid Absorption Rate of Unsaturated Concrete

The moisture diffusivity of concrete is related to the liquid absorption rate. By observing the accumulated length, *l*, of the change in sodium chloride solution level in the horizontal tube of the test, the accumulated fluid absorption amount *i* (per unit area) of unsaturated concrete can be deduced using Equation (1).

(1) $$i=\frac{S_{t}l}{S_{c}}$$

where $S_{t}$ is the cross-sectional area of the horizontal observation tube and $S_{c}$ is the area of the concrete test surface.

**Figure 5:**
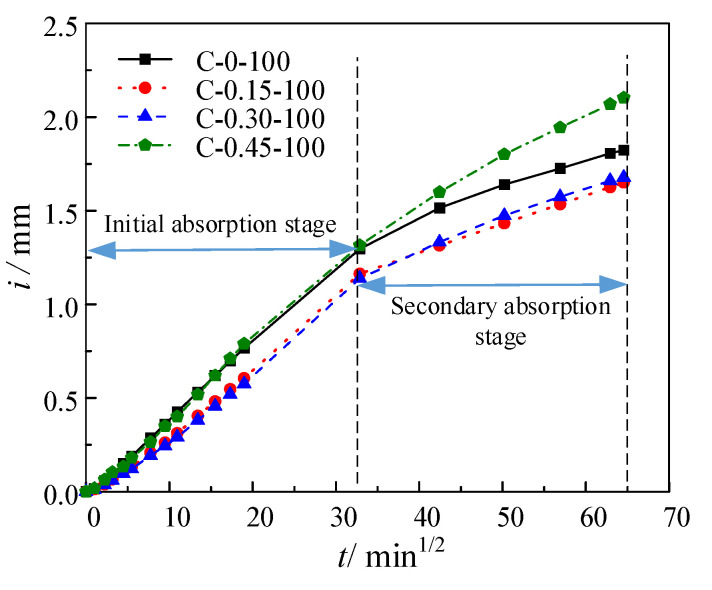
Cumulative liquid absorption of unsaturated concrete under different stress levels.

The liquid absorption rate, is generally defined by means of *i*–*t*^1/2^ [29], where the slope of the *i*–*t*^1/2^ curve is the absorption rate. Taking the test results of coupling loading for 1 million cycles as an example, no matter how the stress level of repeated uniaxial compression loading changes, the *i*–*t*^1/2^ curve of unsaturated concrete is bilinear, corresponding to the initial and secondary liquid absorption stages, respectively, as shown in Figure 5. The demarcation point of the two stages is at about 1080 min (which is 32.86 min^1/2^). Piecewise linear fitting was performed on the *i*–*t*^1/2^ curve. The average liquid absorption rate under each stress condition was obtained. In this test, four groups of tests were carried out for the load conditions with stress levels of 0 and 0.3. In principle, the liquid absorption of concrete under the same load at the same time should be consistent, but due to the randomness of the concrete material, the liquid absorption slightly changes. The influence of the randomness of the concrete material should be removed in the analysis of the data, where the data are averaged. The average liquid absorption of each specimen was calculated at each stage and the cumulative liquid absorption per unit area of concrete was calculated under this stress level. Then, the average liquid absorption rate under each stress condition was obtained by piecewise linear fitting of the averaged curve. The calculation results are shown in Table 4.

The results of the initial and secondary absorption rate of group I in Table 4 show that the initial liquid absorption rate of unsaturated concrete does not change with time, and the secondary liquid absorption rate decays with time. This phenomenon is called the time dependence of the chloride diffusion coefficient of concrete [30,31]. The main reason for this is that the moisture in the concrete increases with the increase in liquid absorption time. The accumulation of the moisture-induced secondary hydration reaction of the cement makes the concrete become denser and reduces the liquid absorption rate. Moreover, with the increase in liquid absorption time, the sodium chloride solution needs to pass through a longer transport channel, which slows down the absorption rate of the concrete. However, the decline in the secondary absorption rate is relatively small under repeated loading.

Through the test results of groups I and III, it can be observed that the secondary absorption rate of C-30%-50 is less than that of C-0-50. The secondary absorption rates of C-30%-100, C-30%-150, and C-30%-200 are higher than C-0-100, C-0-150, C-0-200, respectively. This phenomenon shows that in the early stage of transmission, the new damage inside the concrete with a stress level of 30% is not enough to offset the impact of the initial compaction effect of the test specimens. Thus, the secondary liquid absorption rate is lower than the liquid absorption rate without stress. However, as the loading cycles continue to increase, the damage generated inside the concrete gradually offsets the impact of the initial compaction effect. Fatigue damage creates a channel for chloride transport. The secondary liquid absorption rate is greater than the liquid absorption rate without stress. The initial liquid absorption rate is the liquid absorption rate produced during the rapid water filling process of the capillary pores on the concrete surface. It is related to the porosity of concrete and the initial compression effect of repeated loading. However, the secondary liquid absorption rate is the liquid absorption rate produced during the slow filling of capillary pores in the concrete, which is related to the pores and cracks in concrete. When the loading cycles increase, more concrete damage will occur. The damage increases the connectivity between the pores and accelerates the process of moisture transport.

The uncoupled loading specimen (UC-30%-100) was compared with the coupled loading specimen (C-30%-100), as shown in Table 4. The initial and secondary liquid absorption rates of the uncoupled loading specimens are both larger than the coupled loading specimens. For uncoupled loading specimens, the fatigue loading test was completed before the chloride penetration test. The water transport rate and the liquid absorption rate are both higher for uncoupled loading as well, because the degree of internal crack development is higher before the liquid absorption. Internal damage has accumulated to a relatively severe degree, whereas coupled loading is a gradually developing process. Specimen cracks develop under the combined action of repeated loading and chloride corrosion. The degree of crack development in coupled loading is less than that in uncoupled loading.

### 3.3. Chloride Concentration of Unsaturated Concrete

**Figure 6:**
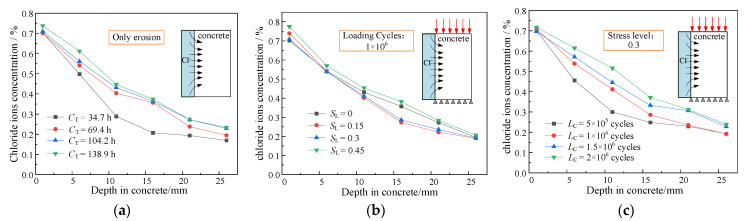
Test results of chloride concentration: (**a**) only erosion; (**b**) different loading cycles; and (**c**) different stress levels.

Figure 6 shows the test results of the chloride concentration of unsaturated concrete specimens at different depths. Under different conditions, as the depth increases, the chloride concentration in the concrete gradually decreases. At depths from the concrete surface between 5 and 20 mm, it can be seen from Figure 6a that the chloride concentration at the same depth changes significantly at corrosion times (CT) between 34.7 h and 69.4 h, but increases only slightly between 69.4 h and 138.9 h. This is mainly related to the saturation degree of the concrete. The surface tension of the contact surface between the chloride solution and concrete causes the chloride to be sucked into the concrete. Chloride moves together with water into the capillary channels of the concrete to balance the pressure difference on both sides of the contacting surface. With the increase in time, when the water content in concrete exceeds the critical value, the chloride concentration difference inside the concrete allows chloride to move from higher to lower concentrations.

The chloride concentration increases first and then decreases with the stress level (*S*_L_) with the same number of loading cycles, as shown in Figure 6b. This shows that the external loads have different impacts on the distribution of the chloride concentration at different stages. When the stress level is less than 0.3, the initial micro-cracks in the unsaturated concrete and the channels between cracks are closed due to compressive stress. The connectivity of the void decreases. This phenomenon is observed and demonstrated as a decrease in the concentration of chloride. When the stress level is greater than 0.3, the micro-cracks in the unsaturated concrete continue to grow and different micro-crack branches are connected. The chloride concentration is observed to increase. As shown in Figure 6c, due to the increase in the loading cycles (*L*_C_), micro-cracks in the concrete expand and gradually connect. The number of channels for the transport and migration of chloride increases, which significantly increases the concentration of chloride at various depths inside the unsaturated concrete.

## 4. Chloride Transport Model of Unsaturated Concrete under Repeated Uniaxial Compressive Loading

### 4.1. Chloride Transport Model

The transport of chloride in unsaturated concrete consists of two stages. In the initial stage of transmission, the moisture content inside the concrete is small. When a solution containing chloride ions reaches the concrete, due to the influence of the liquid surface tension, in order to balance the pressure on both sides of the liquid surface in the capillary channel, chloride ions will be sucked into the concrete together with the moisture. At this point, the transport mode of chloride is convection, which is generated under the action of capillary absorption. With the increase in time, when the moisture content in the concrete exceeds a certain critical value, the chloride in the concrete moves from high concentration areas to low concentration areas under the action of the concentration difference. At this time, the transmission mode of chloride is diffusion. It can be seen that the transport of chloride in unsaturated concrete is the result of diffusion and convection.

Chloride transport in unsaturated concrete is the result of diffusion and convection. Fick’s law has been used to describe the diffusion of chloride [32,33]. The diffusion coefficient is assumed to be proportional to the saturation, *e*, of concrete. The convection flux of chloride is directly proportional to the migration velocity of the moisture in concrete, and the extended Darcy’s law is used to describe the convection velocity of water in unsaturated concrete [34]. The flux of chloride in unsaturated concrete can be expressed as Equation (2):

(2) $$J=-eD_{cl}grad\left(C\right)-CD_{w}\left(\theta \right)\frac{\partial \theta }{\partial x}$$

where *J* is the total flux of chloride in unsaturated concrete (kg/m^2^s); e is the pore saturation of the concrete, i.e., the ratio of the water volume to the pore volume in concrete; $D_{cl}$ is the diffusion coefficient of chloride in concrete (m^2^/s); and C is the concentration of chloride in the concrete pores (kg/m^3^). $D_{w}\left(\theta \right)$ is the moisture diffusion coefficient, and it is expressed as $D_{w}\left(\theta \right)=K\left(\theta \right)d\Psi /d\theta$. $K\left(\theta \right)$ is the hydraulic conductivity, which represents the degree of difficulty for water to pass through the internal pore structure of concrete, which is not a constant but related to the volumetric water content; Ψ is the capillary potential energy; θ is the relative volumetric water content, ranging from 0 to 1; and θ can be calculated by the following Equation (3):

(3) $$\theta =\frac{\Theta -\Theta_{i}}{\Theta_{s}-\Theta_{i}}$$

where $\Theta_{s}$ and $\Theta_{i}$ are the volumetric water content in concrete under saturated and dry conditions, respectively. The initial state of concrete is assumed to be completely dry, namely $\Theta_{i}=0$. It should be noted that a negative value ‘−’ indicates that the permeation of chloride is in the opposite direction of the concentration gradient.

According to the mass conservation law of chloride transport in concrete,

(4) $$\frac{\partial \theta \Theta_{s}C}{\partial t}=-\frac{\partial J}{\partial x}=\frac{\partial }{\partial x}\left[\theta D_{cl}grad\left(C\right)\right]+\frac{\partial }{\partial x}\left[CD_{w}\left(\theta \right)\frac{\partial \theta }{\partial x}\right]$$

the concentration of chloride can be described by the percentage of chloride in the mass of concrete, and the concentration of chloride in pores, C, is converted to the percentage of chloride in the mass of concrete, $C^{\prime }$ [35],

(5) $$C=\frac{C^{\prime }\rho_{c}}{\theta \varphi }$$

By substituting Equation (5) into Equation (4), the chloride transfer model of unsaturated concrete can be expressed as:

(6) $$\frac{\partial C^{\prime }}{\partial t}=\frac{\partial }{\partial x}\left(D_{cl}\frac{\partial C^{\prime }}{\partial x}\right)+\frac{\partial }{\partial x}\left(\frac{C^{\prime }}{\theta }D_{w}\left(\theta \right)\frac{\partial C^{\prime }}{\partial x}\right)$$

The corrosion of the rebaris only related to the content of free chloride. The reason is that the redox reaction cannot occur with bound chloride and reinforcement. Therefore, when considering the effect of bound chloride, the left side of Equation (6) should be the change in total chloride content with time, and the right side should be the free chloride content.

(7) $$\frac{\partial C_{t}}{\partial t}=\frac{\partial \left(C_{f}+C_{b}\right)}{\partial t}=\frac{\partial }{\partial x}\left(D_{cl}\frac{\partial C_{f}}{\partial x}\right)+\frac{\partial }{\partial x}\left(\frac{C_{f}}{\theta }D_{w}\left(\theta \right)\frac{\partial C_{f}}{\partial x}\right)$$

where $C_{t}$ is the total content of chloride, $C_{b}$ is the content of bound chloride, and $C_{f}$ is the content of free chloride.

In this paper, the effect of bound chloride in unsaturated concrete is assumed to be linear. The bound coefficient of chloride is defined as $\lambda =\partial C_{b}/\partial C_{f}$. The transport model considering the bound effect of chloride ions in unsaturated concrete is:

(8) $$\left(1+\lambda \right)\frac{\partial C_{f}}{\partial t}=\frac{\partial }{\partial x}\left[D_{cl}\frac{\partial C_{f}}{\partial x}\right]+\frac{\partial }{\partial x}\left[\frac{C_{f}}{\theta }D_{w}\left(\theta \right)\frac{\partial \theta }{\partial x}\right]$$

In Equation (8), the concentration distribution of free chloride in unsaturated concrete is mainly related to two parameters: the moisture diffusion coefficient, $D_{w}\left(\theta \right)$ and the chloride diffusion coefficient, $D_{cl}$. Additionally, the initial conditions of $C\left(x>0,t=0\right)=C_{0}$ and $C\left(x=0,t>0\right)=C_{s0}$ (boundary condition) should be met. $C_{0}$ is the initial chloride content in the concrete and $C_{s0}$ is the content of chloride on the concrete surface.

Equation (8) contains the concentration of free chloride in concrete, $C_{f}$, and the relative volumetric water content, θ*,* which cannot be solved independently. In the process of water transport, water not only satisfies the extended Darcy’s Law but also satisfies the law of conservation of mass. Therefore, the control equation of capillary water content can be derived in Equation (9):

(9) $$\frac{\partial \theta }{\partial t}=\frac{\partial }{\partial x}\left[D_{w}\left(\theta \right)\frac{\partial \theta }{\partial x}\right]$$

According to the unsaturated seepage theory of capillary absorption in porous media, Equation (9) should meet the initial conditions of $\theta =\theta_{i}$, x≥0, and t=0. The boundary conditions are $\left\{\begin{array}{c} \theta =\theta_{s},x=0,t>0\\ \theta =\theta_{i},x\to \infty ,t>0 \end{array}\right.$, where $\theta_{i}$ is the relative volumetric water content of concrete under dry conditions and $\theta_{s}$ is the relative volumetric water content of concrete in a saturation state.

### 4.2. Moisture Diffusion Coefficient Model

The moisture diffusivity, $D_{w}\left(\theta \right)$, is a nonlinear function of the relative water content. The accuracy of the solution of the moisture transport governing equation depends largely on the accuracy of the moisture diffusion coefficient model. This paper adopted the power function expression [36]:

(10) $$D_{w}\left(\theta \right)=D_{0}\theta^{n}$$

where $D_{0}$ is the parameter of the regression of the experimental data; $D_{0}$ is the peak value of moisture diffusivity and has a decisive effect on the order of magnitude; and *n* is the shape parameter of the diffusion coefficient curve.

Based on work by Parlange et al. [37], the analytical solution of capillary moisture absorption control equation is approximated, and the relationship between the liquid absorption rate and the moisture diffusion coefficient can be expressed as follows:

(11) $$S=\frac{S}{\Theta_{s}-\Theta_{i}}≅{\left[\int_{0}^{1}\left(1+\theta \right)D_{w}\left(\theta \right)d\theta \right]}^{\frac{1}{2}}$$

where *s* is the relative liquid absorption rate and *S* is the liquid absorption rate of the specimen, which is the moisture absorption rate when concrete and water are in contact.

According to Equations (10) and (11), the relationship between $D_{w}\left(\theta \right)$ and the liquid absorption rate, *S*, can be deduced as:

(12) $$D_{w}\left(\theta \right)={\left(\frac{S}{\Theta_{s}-\Theta_{i}}\right)}^{2}\frac{\left(1+n\right)\left(2+n\right)\theta^{n}}{3+2n}$$

Previous studies have shown that the range of the parameter *n* is from 4 to 6 [22,38], and it is not correlated with material properties. Additionally, the smaller the value of *n*, the closer the calculated result is to the experimental result. In view of this, n=4 was chosen in this paper.

According to the test results in Section 3, the liquid absorption rate under repeated uniaxial loading is influenced by the maximum load stress level and loading cycle, as well as the liquid absorption time. In the experimental process, the moisture inside the concrete increases and causes a secondary hydration reaction of the cement. The resulting denser concrete leads to a reduction in the liquid absorption rate. Therefore, the liquid absorption rate of unsaturated concrete under repeated uniaxial compressive loading can be expressed as:

(13) $$S=S_{ref}F_{w}\left(\sigma \right)F_{w}\left(N\right)F_{w}\left(a\right)$$

where *S_ref_* is the liquid absorption rate of the unloaded concrete specimen (mm/min^1/2^); $F_{w}\left(\sigma \right)$ is the influence coefficient of maximum load stress level on the liquid absorption rate; $F_{w}\left(N\right)$ is the influence cofficient of loading cycles on the liquid absorption rate; and $F_{w}\left(a\right)$ is the attenuation coefficient of the liquid absorption rate.

According to Equations (12) and (13), the moisture diffusion coefficient model under repeated uniaxial compressive loading is derived as:

(14) $$D_{w,modified}\left(\theta \right)={\left[\frac{S_{ref}F_{w}\left(\sigma \right)F_{w}\left(N\right)F_{w}\left(a\right)}{\Theta_{s}-\Theta_{i}}\right]}^{2}\frac{\left(1+n\right)\left(2+n\right)\theta^{n}}{3+2n}$$

### 4.3. Chloride Diffusion Coefficient Model

The chloride diffusion coefficient model (Equation (15)) in concrete with the introduction of a crack factor and a fatigue parameter under repeated loading given in reference [35] is as follows:

(15) $$D_{clf}=D_{clm}+0.785D_{clc}\times \frac{N\times \varepsilon_{B}^{P}}{N\times \varepsilon_{B}^{P}+2\times 0.9\times 10^{\left(a-\sigma \right)/b}}$$

where $D_{clm}$ is the chloride diffusion coefficient of the concrete matrix; $D_{ref}$ is the reference value of the chloride diffusion coefficient, considering the water cement ratio and the curing age; $t_{ref}$ is the age at which the specimen is exposed to a chloride corrosion environment; $D_{clc}$ is the chloride diffusion coefficient of the internal concrete cracks, where $D_{clc}=7.5\times 10^{-8}\;m/s^{2}$ according to Yu [35]; *N* is the number of repeated loading cycles; $\varepsilon_{B}^{P}$ is the residual deformation value at the end of the second stage of repeated loading, which is measured to be $\varepsilon_{B}^{P}=1.2\times 10^{-4}$; a and b are test constants, where a=1.07 and b=0.09; and σ is the maximum stress level under repeated loading.

### 4.4. Modification of the Chloride Diffusion Coefficient Model

#### 4.4.1. Influence of the Initial Compression Effect of Uniaxial Compression Load

In the initial stage of repeated loading, the accumulated concrete damage is insignificant. The major influence on the chloride transport at this stage is the initial compression effect of the load on the concrete. When the maximum stress level of the repeated uniaxial compressive loading has not reached the critical value, the chloride diffusion coefficient decreases with the increase in stress level. When the maximum stress level of repeated uniaxial compressive loading is greater than the critical value, the diffusion coefficient of chloride increases with the increase in stress level. Referring to a study by Zhang [39], a quadratic polynomial was selected to describe the influence of initial compression under repeated uniaxial loading on the chloride diffusion coefficient, where the influence coefficient is:

(16) $$F_{cl}\left(\sigma \right)=a_{1}\sigma^{2}+b_{1}\sigma +c_{1}$$

where $a_{1}$, $b_{1}$, and $c_{1}$ are empirical coefficients.

#### 4.4.2. Influence of the Curing Load

The curing age will also affect the transport of chloride in the concrete. Longer curing times provide sufficient time for the hydration reaction of the cement inside the concrete, resulting in fewer and smaller internal pores. Less and smaller internal pores mean fewer channels for chloride and moisture transport, resulting in a reduced diffusion coefficient. The influence of curing age on the diffusion coefficient of chloride [35] is:

(17) $$F_{cl}\left(c\right)={\left(\frac{t_{c}}{t_{c0}}\right)}^{-0.1}$$

where $t_{c}$ is the actual curing age of the concrete and $t_{c0}$ is the curing age for a reference value of the chloride diffusion coefficient, generally 28 days.

#### 4.4.3. Time-Dependent Influence of the Matrix Diffusion Coefficient

The diffusion coefficient of the concrete matrix decreases with the increase in chloride ion erosion time. The reason for this is that with the increase in time, the cement inside the concrete undergoes a secondary hydration reaction, which makes the concrete more compact. The transmission path of chloride ions is reduced, so the chloride ion diffusion coefficient will continue to decrease:

(18) $$D_{clm}=D_{ref}{\left(\frac{t_{ref}}{t}\right)}^{3\times \left(0.55-w/c\right)}$$

Therefore, the influence of the initial compression effect of repeated uniaxial compressive loading, $F_{cl}\left(\sigma \right)$, the influence of the curing age on chloride diffusion coefficient $F_{cl}\left(c\right)$, and the concrete matrix time-dependent diffusion coefficient, $D_{clm}$, have been introduced. The chloride diffusion coefficient, considering repeated loading, is now:

(19) $$D_{clf,modified}=F_{cl}\left(\sigma \right)F_{cl}\left(c\right)\left[D_{clm}+0.785D_{clc}\times \frac{N\times \varepsilon_{B}^{P}}{N\times \varepsilon_{B}^{P}+2\times 0.9\times 10^{\left(a-\sigma \right)/b}}\right]$$

## 5. Analysis and Discussion of Chloride Transport in Unsaturated Concrete

### 5.1. Recommended Liquid Absorption Rate Calculation Method

**Figure 7:**
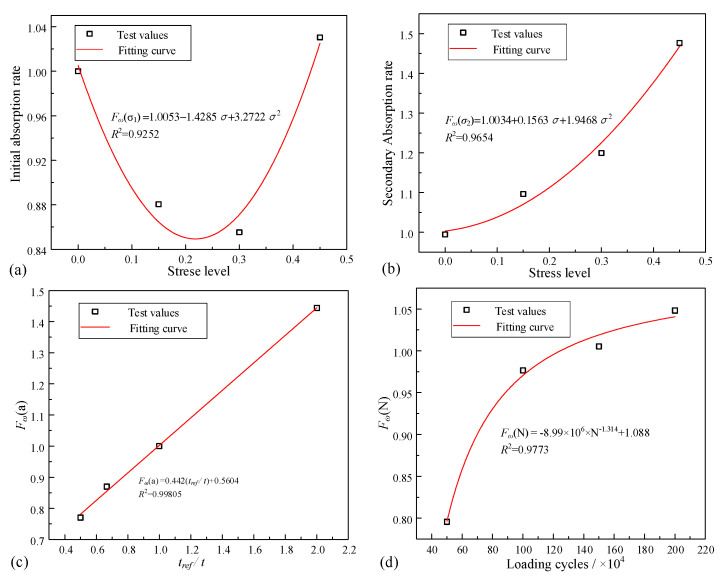
Moisture transport influence factor fitting: (**a**) initial absorption stress level; (**b**) secondary absorption stress level; (**c**) *F_ω_*(a) vs. *t_ref_*/*t*; and (**d**) *F_ω_*(N) vs. loading cycles.

From the test results in Section 3.2, it can be observed that the initial liquid absorption rate of unsaturated concrete is related to the compaction effect. The compaction effect is affected by the stress level. The secondary liquid absorption rate is influenced by the stress level and the number of loading cycles, as well as the attenuation coefficient of the secondary liquid absorption rate. Taking the initial and secondary liquid absorption rates of C-0-100 control specimens as the standard values, the liquid absorption rates under various working conditions were normalized, and the calculation expressions of each influence factor of the moisture diffusion coefficient were fitted. The fitting curves are shown in Figure 7.

According to the above analysis, the suggested correction coefficient is substituted into Equation (10). The relationship between the load factor of the repeated uniaxial compressive loading and the liquid absorption rate of the unsaturated concrete is

(20) $$S=\left\{\begin{array}{cc} 0.0402, & \;\;t\leq 1080\;\min\\ 0.0162\times \left[0.442\left(\frac{t_{ref}}{t}\right)+0.5604\right], & \;\;t\geq 1080\;\min \end{array}\right.$$

when σ=0, and

(21) $$S=\left\{\begin{array}{c} \;\;\;\;\;\;\;\;\;\;\;\;\;\;\;\;\;\;\;\;\;\;\;0.0402\times \left(1.0053-1.4285\sigma +3.2722\sigma^{2}\right),\;\;\;\;\;\;\;\;\;\;\;\;\;\;\;\;\;\;\;\;\;\;\;\;\;t\leq 1080\;\min\\ 0.0162\times \left[0.442\left(\frac{t_{ref}}{t}\right)+0.5604\right]\times \left(1.0053-1.4285r+3.2722r^{2}\right)\times \;\;\;\;\;\;\;\;\;\;\;\;\;\\ \left(1.0034+0.1563r+1.9468r^{2}\right)\times \left[-8.99\times 10^{6}\times {\left(N\right)}^{-1.314}+1.088\right],\;\;t>1080\;\min \end{array}\right.$$

when σ≠0

### 5.2. Distribution of Relative Volumetric Water Content

**Figure 8:**
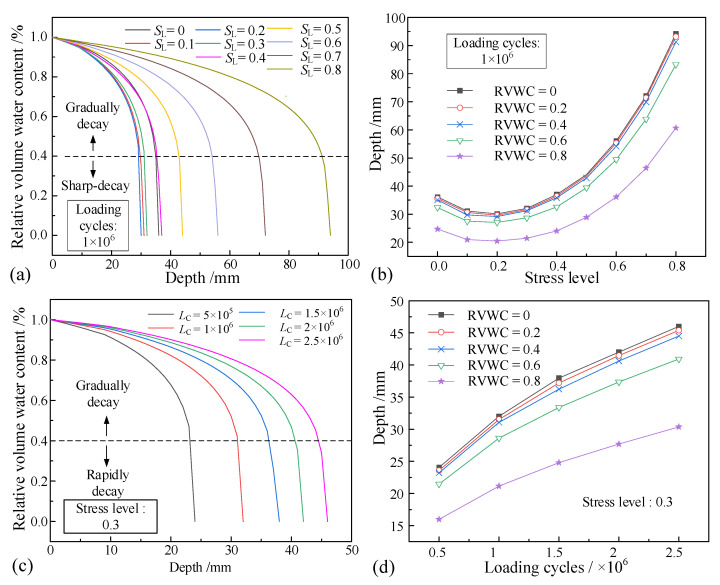
Relative water content distribution (**a**); stress level at 1 × 10^6^ loading cycles (**b**); relative volume water content (**c**); and (**d**) loading cycles under 0.3*σ*_max_.

According to the concrete capillary water control equation (Equation (6)) and its approximate analytical solution, the software Matlab was used to calculate the distribution of relative volumetric water content in unsaturated concrete. The relative volumetric water absorption of unsaturated concrete under different stress levels and different loading cycles was calculated by using the average liquid absorption in each stage. The solutions are shown in Figure 8.

As shown in Figure 8a,c, the relative volumetric water content (RVWC) decreases with increasing depth inside each specimen. The change in the relative volume water content can be divided into two stages: the gradual decay stage and the rapid decay stage. The gradual decay stage takes place from 1.0 to 0.4. With the increase in penetration depth, the relative volumetric water content decreases slowly. When the water content is 0–0.4, the rapid decay stage takes place. At this stage, the relative water content drops sharply to 0. There is little difference between the total penetration depth and the relative volumetric water content of 0.4. Therefore, the penetration depth at the relative volumetric water content of 0.4 can be selected as the reference penetration depth.

When the relative volumetric water content is the same, the stress level has a great influence on the penetration depth, as shown in Figure 8b. As the stress level increases, the penetration depth shows a trend of first decreasing and then increasing. The minimum value is reached at the stress level of 0.2. The initial compaction effect is greater than the damage accumulation caused by fatigue. When the stress level is greater than 0.2, the fatigue damage accumulation increases gradually as the penetration depth increases. As shown in Figure 8d, with the increase in the loading cycles, the relative volume of water content in the unsaturated concrete continues to increase, indicating that the internal damage of concrete caused by repeated loading has a significant impact on the moisture penetration of unsaturated concrete. Corresponding to the fatigue characteristics of concrete, the growth becomes slower. In conclusion, the established model can accurately calculate the liquid absorption of concrete under repeated loading.

### 5.3. Distribution of Chloride Concentration

Based on the water diffusion coefficient model fitted by the test data and the modified chloride diffusion coefficient model, the Crank–Nicolson finite difference method and Thomas algorithm were used to solve the chloride transport model of unsaturated concrete under repeated uniaxial compressive loading (Equation (5)). It is assumed that the initial compression effect of uniaxial compression repeated loading has the same influence on the transport behavior of both water and chloride, namely $F_{cl}\left(\sigma \right)=F_{\omega }\left(\sigma_{1}\right)$.

**Figure 9:**
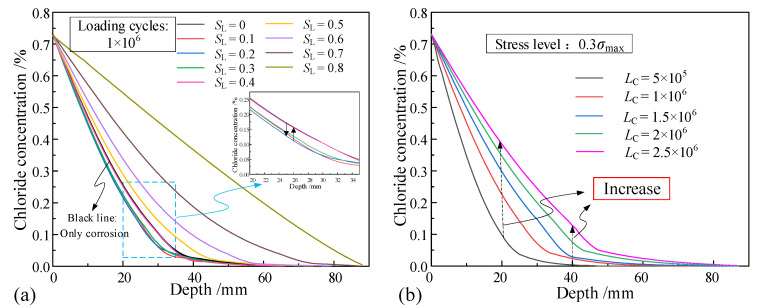
Chloride concentration in unsaturated concrete: (**a**) effect of different repeated loading stress levels and (**b**) effect of different repeated loading cycles.

The chloride concentration distribution in unsaturated concrete under different stress levels and loading cycles was calculated according to the proposed model, as shown in Figure 9. Under the same amount of loading cycles, with the increase in stress level, the chloride concentration first decreases and then increases. When the stress level is greater than 0.2, the chloride content in the same position in the concrete increases continuously as the stress level increases. The initial micro-cracks in the unsaturated concrete and the connecting channels between the cracks are closed under the action of pressure when the stress level is less than 0.2. When the stress level is greater than 0.2, the micro-cracks in the unsaturated concrete extend and connect, and the transport channels of chloride increase. Therefore, the transport rate of chloride accelerates gradually. The propagation rate of micro-cracks in the unsaturated concrete increases with the increase in the stress level. This phenomenon also can be observed in [20]. However, in contrast to the results in [9], when the stress level is smaller than 0.2, the chloride concentration decreases. The conclusion is that the damage caused by the smaller stress levels on the concrete is less significant than its compaction effect on the internal pores of the concrete. When the stress level is greater than 0.2, the damage to the concrete increases with the increase in stress levels. Under the effects of convection and diffusion, more chloride enters the concrete and the chloride concentration at the same depth increases.

As shown in Figure 9b, at the same stress level, the internal chloride penetration depth and concentration both increase with the increase in loading cycles. Corresponding to the three-stage characteristics of fatigue, in the initial stages of repeated loading, internal cracks in the unsaturated concrete intensively develop and the transport rate of chloride is fast. In the middle stages of repeated loading, the development of the cracks in the unsaturated concrete enters a stable stage. Moreover, as the propagation rate of the cracks slows down, the transport rate of chloride slows down accordingly. The last stage is the unstable fracture propagation stage, which is on the verge of structural failure and is not the focus of this study.

### 5.4. Comparative Analysis of Chloride Transport in Unsaturated and Saturated Concrete

**Figure 10:**
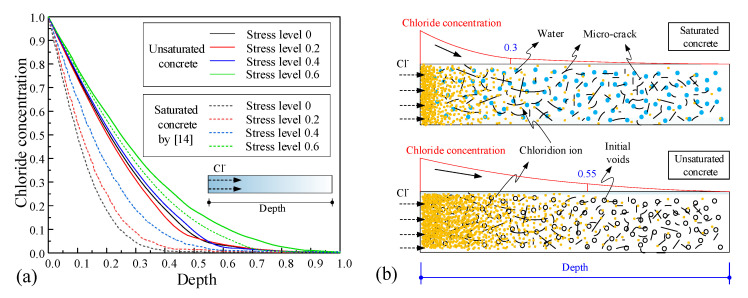
Chloride transport in unsaturated and saturated concrete under repeated loading: (**a**) normalized comparison and (**b**) schematic diagram.

To analyze the influence of the state of concrete on chloride transport, a normalized chloride transmission law of saturated concrete from the literature [20] was compared with the results of unsaturated concrete in this paper, as shown in Figure 10. Under the coupled action, the chloride concentration in unsaturated concrete is significantly higher than that in saturated concrete at the same stress level. After the concrete is saturated with water, the initial internal pores and micro-cracks are filled with water, and chloride can only travel through the damage channels generated by repeated loading. At the same time, the solution entering the damaged channels is diluted by the presence of free water in the saturated concrete, resulting in a lower chloride concentration.

The transport depth of chloride in unsaturated concrete is significantly larger than that in saturated concrete, as shown in Figure 10. When the normalized corrosion depth is greater than 0.3, the chloride concentration in saturated concrete is less than 0.1. When the normalized corrosion depth of unsaturated concrete is greater than 0.55, the chloride concentration becomes insignificant. The corrosion depth of unsaturated concrete is roughly twice of the saturated concrete, and the concentration distribution is more uniform, as shown in Figure 10b. Therefore, compared with saturated concrete, the chloride concentration and corrosion depth of unsaturated concrete are both higher under repeated loading. The corrosion effect of chloride is more pronounced for unsaturated concrete.

## 6. Conclusions

This paper conducted an experimental study on the transport of chloride inside unsaturated concrete under repeated loading. Based on the experimental results, a theoretical model for the calculation of chloride transport inside the unsaturated concrete under repeated uniaxial compressive loading was proposed. The main conclusions can be summarized as follows:

1. Under repeated loading, the liquid absorption rate of unsaturated concrete exhibits an obvious two-stage trend. The liquid absorption rate of unsaturated concrete in the initial stage is mainly related to the stress level in capillary action and repeated loading. The increase in the liquid absorption rate in the second stage is mainly caused by diffusion of chloride through the accumulated damage channels inside the concrete under repeated loading.
2. The internal damage of concrete before chloride exposure leads to changes in the transmission channels of moisture and chloride in the uncoupled test method. Thus, the penetration of moisture and chloride in unsaturated concrete will be overestimated.
3. Fully considering the coupled effect of repeated loading and corrosion, the chloride diffusion coefficient and water diffusion coefficient are modified. The chloride transport model of unsaturated concrete under uniaxial compressive repeated load is established.
4. The penetration depth when the relative moisture content is 0.4 can be used as the critical penetration depth of chloride for unsaturated concrete under coupled action. Repeated loading with a stress level of less than 0.2 will improve the chloride resistance of unsaturated concrete.
5. The chloride transport in unsaturated and saturated concrete is different under the coupled action of repeated loading and corrosion. Unsaturated concrete has a higher chloride concentration and corrosion depth. The effect of chloride is more pronounced in unsaturated concrete.

## Figures and Tables

**Table 1:** Mix proportions of concrete.

Water/Cement Ratio	Mix Proportions (kg/m^3^)						
	Cement	Slag Powder	Fly Ash	Fine Aggregate	Coarse Aggregate	Water	Admixture
0.32	212	212	47	724	1041	150	4.7

**Table 2:** Statistical table of repeated loading conditions under uniaxial compression.

Specimen No.	Loading Method	*σ*_max_	*σ*_min_	Corrosion Time /Loading Cycles	Frequency
C-0-50	—	—		34.7 h	—
C-0-100				69.4 h	
C-0-150				104.2 h	
C-0-200				138.9 h	
C-0.15-100	Coupled	0.15*σ*_max_	0.05*σ*_max_	1 × 10^6^ cycles	4 Hz
C-0.3-50		0.3*σ*_max_		5 × 10^5^ cycles	
C-0.3-100		0.3*σ*_max_		1 × 10^6^ cycles	
C-0.3-150		0.3*σ*_max_		1.5 × 10^6^ cycles	
C-0.3-200		0.3*σ*_max_		2 × 10^6^ cycles	
C-0.45-100		0.45*σ*_max_		1 × 10^6^ cycles	
UC-0.3-100	Uncoupled	0.3*σ*_max_		1 × 10^6^ cycles	

Notes: C is for coupled loading; UC is for uncoupled loading. Specimens with a stress of 0 are control specimens, and the corrosion time is the time corresponding to each loading cycle. The time taken was 34.7 h, 69.4 h, 104.2 h, and 138.9 h for 0.5 million, 1 million, 1.5 million, and 2 million cycles of loading, respectively.

**Table 3:** Measurement time and allowable time error.

Time	60 s	5 min	10 min	20 min	Record Every 30 min before 2 h	Record Every 30 min before 6 h	Record Every 12 h before 90 h	Record Every 24 h
Allowable time error	2 s	10 s	2 min	2 min	2 min	5 min	2 h	2 h

**Table 4:** Liquid absorption statistics of unsaturated concrete specimens.

Test Group	Specimen No.	Initial Absorption Stage		Secondary Absorption Stage	
		Initial Absorption Rate	Correlation Coefficient	Secondary Liquid Absorption Rate	Correlation Coefficient
I	C-0-50	0.0402	0.9987	0.0234	0.9989
	C-0-100			0.0162	0.9792
	C-0-150			0.0141	0.9772
	C-0-200			0.0125	0.9494
II	C-15%-100	0.0354	0.9929	0.0153	0.9929
III	C-30%-50	0.0344	0.9891	0.0199	0.9988
	C-30%-100			0.0169	0.9933
	C-30%-150			0.0149	0.9838
	C-30%-200			0.0142	0.9797
IV	C-45%-100	0.0414	0.9969	0.0245	0.9923
V	UC-30%-100	0.0521	0.9970	0.0256	0.9911

## Data Availability

The data presented in this study is available on request from the corresponding author.
